# Supplementary material for: Immunocapture Magnetic Beads Enhanced the LAMP-CRISPR/Cas12a Method for the Sensitive, Specific, and Visual Detection of Campylobacter jejuni
Source: Biosensors (Basel). 2022 Mar 2;12(3):154. doi: 10.3390/bios12030154 (PMC8946501; doi:10.3390/bios12030154)
Supplement: Supplementary file 1 [file biosensors-12-00154-s001.zip › biosensors-1592712-supplementary.pdf]

Supplementary

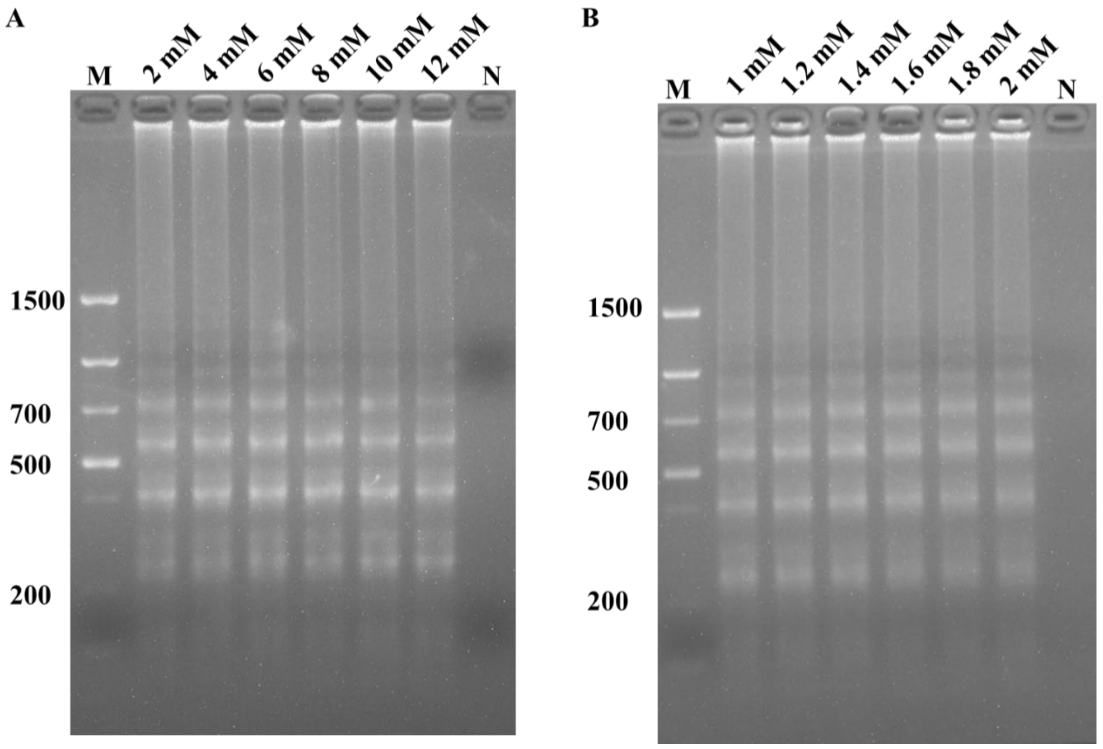

Figure S1 Evaluation of Mg^{2+} and dNTPs in the LAMP reaction.

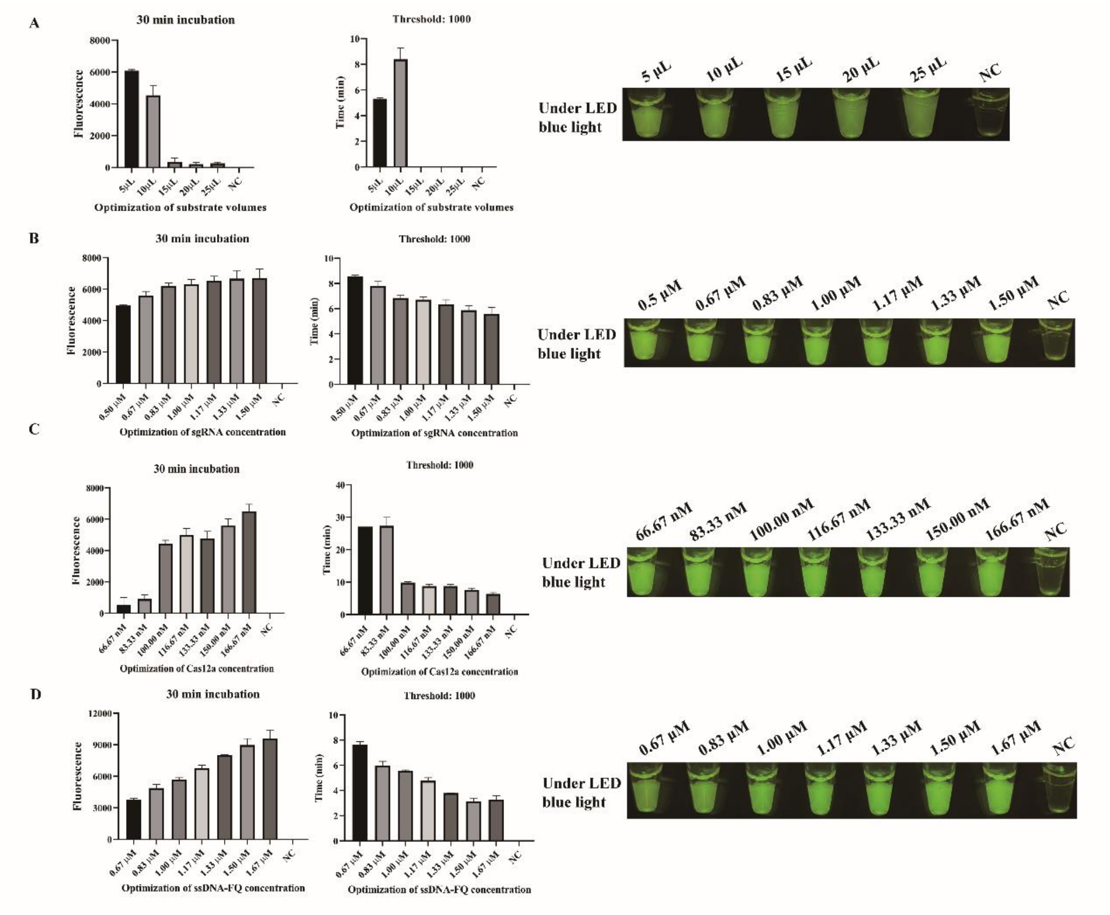

Figure S2 Evaluation of the concentration of sgRNA, Cas12a, the ssDNA-FQ probe, and the substrate volume.

Table S1. Average binding efficiency of ICB.

Initial concentration (CFU/mL)	20 min after capture (CFU/mL)					Binding efficiency (%)	Average binding efficiency (%)
	1	2	3	Mean value	Difference before and after the capture		
$8*10^3$	1220	1430	1310	1320	7220	84.5	
$8*10^2$	40	80	70	63.3	790.7	92.6	92.4
$8*10^1$	0	0	0	0	80	100	

Table S2 The sequence information of all used primers, sgRNAs, and the ssDNA probe

Name	Sequence (5'-3')	Length (bp)
1F3	GCGCAAAAATTAAAAGAATTTGG	23
1B3	GCAGCAAGCAATAAAGAAGT	20

1FIP	GCACGAAGTCCTATTTTTTTATCGCTTTTGTTTATGAGGAAATAGGAAAAACA G	54
1BIP	ACAAGAATGCACAAATTTGCCTTTTTTTCCATCATGACCACAAGC	45
2F3	GAAAAACAGGCGTTGTGG	18
2B3	CCTCTTCAGCAGGTTGAA	18
2FIP	GCATTCTTGTAAAGGCAAAGCATTTTTGGAAATAGCGATAAAAAAATAGGAC	52
2BIP	TGCATGCTTGTGGTCATGATGTTTTCGCCATTAAAATTCTGACTTG	46
3F3	TTTGGTTATGAGGTTTATGAGG	22
3B3	GCAGCAAGCAATAAAGAAGT	20
3FIP	CCATATCTGCACGAAGTCCTATTTTTTTTAAATAGGAAAAACAGGCGTT	49
3BIP	ACAAGAATGCACAAATTTGCCTTTTTTTCCATCATGACCACAAGC	45
4F3	GGCGAATTTGAAAAAATTCGTC	22
4B3	GCATCCATATCTGCACGA	18
4FIP	TGCGCCACTAATTTTGCAGTACTTTTATCAAATTCATGAAAATCCTGAGC	50
4BIP	GAGGAAATAGGAAAAACAGGCGTTTTTCCTATTTTTTTATCGCTATTTCCCT	52
5F3	GATGATGGCTTCTTCGGATA	20
5B3	TGATATTAAAAGCATGTCCTGC	22
5FIP	AGCAGCATAAATAGGATCTTTTGCCTTTTTATTGAAGTTATTGGAAGGGGT	51
5BIP	TGTGGCTTTACAAAGCATAGTATCTTTTTAGCTCCTATGCTTACAACTG	49
sgRNA1	GAAUUUCUACUGUUGUAGAUCAAGAAUGCACAAAUUUGCCUUAU	44
sgRNA2	GAAUUUCUACUGUUGUAGAUUUUUUUUGCUUUUAUAAGGCAAAUU	44
sgRNA3	GAAUUUCUACUGUUGUAGAUCAGCAAGCAAUAAAGAAGUAGUAU	44
sgRNA4	GAAUUUCUACUGUUGUAGAUCCUUUACAAGAAUGCACAAAUUUG	44
sgRNA5	GAAUUUCUACUGUUGUAGAUUAAGGCAAAUUUGUGCAUUCUUGU	44
sgRNA6	GAAUUUCUACUGUUGUAGAUCUUUUAUAAGGCAAAUUUGUGCAU	44
ssDNA-FQ probe	FAM-TTTTTT-BHQ1	6
